# ARTD-Net: Anchor-Free Based Recyclable Trash Detection Net Using Edgeless Module

**DOI:** 10.3390/s23062907

**Published:** 2023-03-07

**Authors:** BoSeon Kang, Chang-Sung Jeong

**Affiliations:** 1 Visual Information Processing, Korea University, Seoul 02841, Republic of Korea; 2 Department of Electrical Engineering, Korea University, Seoul 02841, Republic of Korea

**Keywords:** object detection, waste management, recyclable waste dataset, deep neural networks

## Abstract

Due to the sharp increase in household waste, its separate collection is essential in order to reduce the huge amount of household waste, since it is difficult to recycle trash without separate collection. However, since it is costly and time-consuming to separate trash manually, it is crucial to develop an automatic system for separate collection using deep learning and computer vision. In this paper, we propose two Anchor-free-based Recyclable Trash Detection Networks (ARTD-Net) which can recognize overlapped multiple wastes of different types efficiently by using edgeless modules: ARTD-Net1 and ARTD-Net2. The former is an anchor-free based one-stage deep learning model which consists of three modules: centralized feature extraction, multiscale feature extraction and prediction. The centralized feature extraction module in backbone architecture focuses on extracting features around the center of the input image to improve detection accuracy. The multiscale feature extraction module provides feature maps of different scales through bottom-up and top-down pathways. The prediction module improves classification accuracy of multiple objects based on edge weights adjustments for each instance. The latter is an anchor-free based multi-stage deep learning model which can efficiently finds each of waste regions by additionally exploiting region proposal network and RoIAlign. It sequentially performs classification and regression to improve accuracy. Therefore, ARTD-Net2 is more accurate than ARTD-Net1, while ARTD-Net1 is faster than ARTD-Net2. We shall show that our proposed ARTD-Net1 and ARTD-Net2 methods achieve competitive performance in mean average precision and F1 score compared to other deep learning models. The existing datasets have several problems that do not deal with the important class of wastes produced commonly in the real world, and they also do not consider the complex arrangement of multiple wastes with different types. Moreover, most of the existing datasets have an insufficient number of images with low resolution. We shall present a new recyclables dataset which is composed of a large number of high-resolution waste images with additional essential classes. We shall show that waste detection performance is improved by providing various images with the complex arrangement of overlapped multiple wastes with different types.

## 1. Introduction

Recently, the increasing population has been accompanied by a vast amount of resource consumption. Therefore, separate collection is essential to reduce the huge amount of household waste by recycling trash. Since it is costly and time-consuming to separate trash manually, it is very important to develop an automatic system for separate collection using deep learning and computer vision [1,2,3,4]. In previous research on automatic systems for separate collection, many methods have been proposed for trash classification using deep learning and computer vision [5,6,7]. However, they only deal with the simple case for the image with a single trash. In order to overcome the limitations of single garbage classification, previous research has worked on methods for detecting multiple types of trash [8,9]. However, the trash datasets used in their research have several problems which make it difficult for deep learning models to detect trash in the real world. First, the existing dataset does not efficiently deal with various wastes of the real world, since it does not include important types of waste such as cans and PETs. Second, since the existing dataset is used for classifying single waste in one image, it does not consider data with the complex arrangement, i.e., with several overlapped wastes with different types. Third, most of the existing datasets have a small number of data with low resolution. In order to resolve those problems, we are concerned with the design of deep learning based model for recognizing multiple wastes of different types in the complex image which may be overlapped as well as the creation of large-scale high-resolution dataset with various types.

In this paper, we shall propose two Anchor-free based Recyclable Trash Detection Networks (ARTD-Net) for accurately recognizing overlapped multiple wastes of different types by using edgeless module: ARTD-Net1 and ARTD-Net2. The former is an anchor-free-based one-stage deep learning model which consists of three modules: centralized feature extraction, multiscale feature extraction and prediction. The centralized feature extraction module is used as the backbone in the model, focusing on extracting features around the center of the input image. The extracted feature maps are passed on to the multiscale feature extraction module for further processing. The multiscale feature extraction module generates five feature maps of different sizes through the combination of bottom-up and top-down pathways. The prediction module improves detection accuracy by exploiting feature maps of different sizes and efficiently finding small-sized objects using anchor-free detection, and adjusting edge weights for each instance to improve classification accuracy of multiple objects. The latter is an anchor-free based multi-stage deep learning model which can efficiently finds each of waste regions by additionally exploiting Region Proposal Network and RoIAlign. It sequentially performs classification and regression to improve accuracy. Additionally, we build a new recyclables dataset which consists of a large number of high-resolution images of waste. Our dataset provides various images with complex arrangements, such as overlapped wastes of different types, and comprises additional essential classes.

The contributions of our research are summarized as follows:

1. We propose two Anchor-free based Recyclable Trash Detection Networks (ARTD-Net) which can recognize overlapped multiple wastes of different types efficiently by using edgeless module: ARTD-Net1 and ARTD-Net2. ARTD-Net2 is more accurate than ARTD-Net1, while ARTD-Net1 is faster than ARTD-Net2. We shall show that our proposed ARTD-Net1 and ARTD-Net2 achieve competitive performance in mean average precision and F1 score compared to other deep learning models.
2. We propose a background weight adjustment block in the centralized feature extraction module which improves the detection accuracy by focusing on around the center of the input image based on centralized weights adjustments.
3. We propose an object instance separation block in the prediction module which improves the classification accuracy of multiple objects based on edge weights adjustments for each instance.
4. We present a multi-stage model for ARTD-Net which efficiently finds each of waste regions by using Region Proposal Network and RoIAlign.
5. We contribute a new large scale recyclables dataset which comprises various additional essential classes, and include various high resolution images with the complex arrangement, i.e., with several overlapped wastes with different types.

The remainder of this paper comprises four sections. In Section 2, we review studies on waste classification and anchor-free models. In Section 3, we present the overall architecture for our waste detection model. In Section 4, we describe our experimental results, and finally in Section 5 we conclude with our results.

## 2. Related Work

### 2.1. Trash Dataset

#### 2.1.1. Classification

TrashNet dataset [10] is a collection of 2527 images of six classes of waste, including trash, metal, plastic, glass, cardboard and paper. A number of subsequent studies have been conducted with the TrashNet dataset [11]. RecycleNet achieved 81% accuracy on the Trashnet dataset. The best result was achieved with the DenseNet121 [12] model. They changed the connection pattern between high-density blocks and reduced the parameters of the 121-layer network from 7 million to approximately 3 million in order to compensate for the slow prediction time [13]. Ruiz et al. achieved an accuracy of 88.6% based on the ResNet model [14] after adding non-waste class to the TrashNet dataset. They experimented with CNN [15], VGG [16] and Inception for automatic garbage classification [17]. The DNN-TC model is based on the ResNext architecture and applies several preprocessing techniques. Two fully connected layers were added to reduce redundancy and improve performance. In addition, they collected inorganic, organic, and medical waste from Vietnam and included them in the dataset. The performance of DNN-TC model was compared with that of the existing TrashNet dataset, and the results were analyzed [18].

#### 2.1.2. Segmentation

The TACO dataset was created to detect waste dumped in the sea. This dataset consists of 1500 images with 4,784 annotations that are labeled in 60 subcategories that belong to 28 super-categories [19]. MJU-Waste dataset was built to compensate for the shortcomings of the TACO dataset. It consists of 2475 RGBD images taken with Microsoft Kinect RGBD camera. They improved detection accuracy by applying the intensity and depth information to multiple levels of spatial granularity to previous deep learning models. The model with ResNet-101 as the backbone achieved an Intersection over Union (IoU) score of 87.84 [20].

### 2.2. Object Detection Method

Object detection methods can generally be divided into two main methods: one-stage and multi-stage. One-stage method performs classification and regression at the same time. This results in faster processing times compared to the multi-stage method. We analyze YOLO series [21,22,23,24,25,26], SSD [27], FASF [28], FCOS [29], SABL [30], SOLO [31], CornerNet [32], and CentripetalNet [33] and compare their performance with ARTD-Net1. The multi-stage method performs regression and classification sequentially. It is more accurate than one-stage method. We analyze Faster R-CNN [34], Cascade R-CNN [35], Double-Head RCNN [36], Sparse R-CNN [37] and DetectoRS [38] and compare their performance with ARTD-Net2.

YOLO is a real-time object detector designed for high-speed processing. It finds the location and class using a single network pass. YOLO divides the input image into multiple grids, where the grid cell closest to the center of the object is responsible for detecting the object. Each grid cell predicts the corresponding bounding boxes, along with their confidence scores and conditional class probabilities. YOLO has a limitation where the performance is somewhat reduced for small objects due to the small difference in IoU values. To address this limitation, improved models such as YOLOx, YOLOF, YOLOv5 and YOLOv6 have been proposed.

SSD detects objects of various sizes by dividing the input image into grids of different sizes across six feature maps. It is constructed using six additional convolution layers on the fifth convolution layer of VGG-16.

FSAF is a one-stage model based on RetinaNet [39]. They improved the performance by using multi-level anchor-free branches which solved the problems of the existing anchor-based model. During training, anchor-free branches attached to each level of the Feature Pyramid Network (FPN) [40] select the most appropriate feature level for training, which effectively represents the instance. As a result of training, the model achieves better performance than existing one-stage detectors in detecting small objects.

FCOS is a one-stage model which detects objects in a per-pixel prediction fashion. It is based on an anchor-free method and reduces training time by eliminating the need for complex calculations associated with anchor boxes such as calculating overlapping during training. They utilize centerness to decrease the impact of bounding boxes generated far from the center of an object. Centerness reduces the influence of predicted values at locations far from the center of the object using the center of the bounding box, right-bottom, left-top and corner pairs.

CornerNet predicts object bounding boxes using a keypoint pair instead of anchors, where the keypoints represent the top-left and top-right corners of the target object. Instead of using anchor boxes, CornerNet estimates these keypoints based on feature points. CentripetalNet is a keypoint-based object detector which utilizes centripetal shift to generate a keypoint pair representing the corners of the same object.

Faster R-CNN is an object detection model which is developed to improve the slow speed of R-CNN caused by selective search. The selective search is the slowest part of R-CNN. It is computed in the CPU. To improve this, Faster R-CNN introduces RPN that can perform computations on the GPU. RPN takes a feature map as input which is obtained from the previous convolutional neural network layer. A 256-dimension vector is obtained using a sliding window on the received feature map. At this time, the anchor to be used as the window being set in advance. In Faster R-CNN, nine anchors with various width, height, ratio, and size are used. The class and location of the object are calculated through two layers using the 256-dimensional vector obtained in this way.

Cascade R-CNN proposes a method to address two issues that occur with the increase in IoU thresholds. With an increase in IoU thresholds, the number of positive samples decreases exponentially, which can lead to overfitting during training. In addition, if there is a difference between IoU thresholds used during training and those used at inference, it can result in decreased accuracy. Cascade R-CNN is comprised of a sequence of detectors with different IoU thresholds set. Detectors are connected sequentially and use the output from the previous step as input for the next step. As a result, each detector has the positive set of examples of equivalent size to solve the overfitting problem. It shows that performance is improved through the process of gradual training using the proposals of the learned detectors at low IoU.

The Double-Head method utilizes the commonly used two head structures for classification and localization tasks in R-CNN based detectors. They found that the fully connected head is better for classification while the convolution head is better for localization. To leverage the strengths of both structures, they proposed the Double-Head method which combines a fully connected head for classification and a convolution head for bounding box regression.

Sparse R-CNN is a purely sparse method for object detection. It utilizes a fixed sparse set of learned object proposals provided to the object recognition head for classification and location. Sparse R-CNN directly outputs final predictions without a non-maximum suppression post-procedure and demonstrates accuracy performance on par with well-established detector baselines on the COCO dataset.

DetectoRS proposes a backbone design utilizing a see and think mechanism with improvements at two levels: macro and micro. At the macro level, the method involves building Recursive Feature Pyramid by adding feedback connections to the existing FPN. At the micro level, they propose Switchable Atrous Convolution which utilizes a switch function to collect features extracted at various atrous rates.

## 3. Model Architecture

In this section, we shall present ARTD-Net which is an anchor-free based deep learning model for detecting multiple wastes with various types. We propose two versions of ARTD-Net: ARTD-Net1 and ARTD-Net2. ARTD-Net1 is a one-stage model which performs classification and regression at the same time, while ARTD-Net2 a multi-stage model which sequentially performs classification and regression. ARTD-Net1 is faster than ARTD-Net2, but ARTD-Net2 is more accurate than ARTD-Net1.

### 3.1. ARTD-Net1

**Figure 1:**
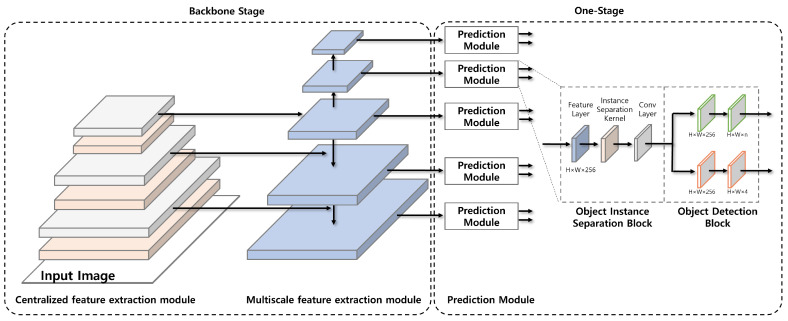
ARTD-Net1.

As shown in Figure 1, ARTD-Net1 is an anchor-free-based one-stage model which consists of three modules: centralized feature extraction, multiscale feature extraction and prediction. The centralized feature extraction module is used as backbone in the model. It focuses on extracting features around the center of the input image. It consists of three layers each of which extracts a feature map of different size. The feature map from each layer is sent to multiscale feature extraction module. The multiscale feature extraction module is composed of five layers each of which produces a feature map of different size. The feature map in each layer is sent to prediction module. Each of five prediction modules finds the region and class for multiple wastes of different size based on the feature map from multiscale feature extraction module.

#### 3.1.1. Centralized Feature Extraction Module

The centralized feature extraction module is obtained by adding BWAB (Background Weight Adjustment Block) to RetinaNet. BWAB exploits a new scheme which efficiently extracts features around the center of the input image while reducing the edge weights around the border of the image. BWAB consists of two components: Background Weight Adjustment kernel and the convolution layer. Let $F_{i}\in R^{H\times W\times C}$ be the feature maps at layer *i* of a backbone, and *H* and *W* the height and width of the feature map, respectively, and *C* the number of class. Let (x,y) be the coordinates of the feature map, x∈X={0,1,…,W},y∈Y={0,1,…,H}.

**Figure 2:**
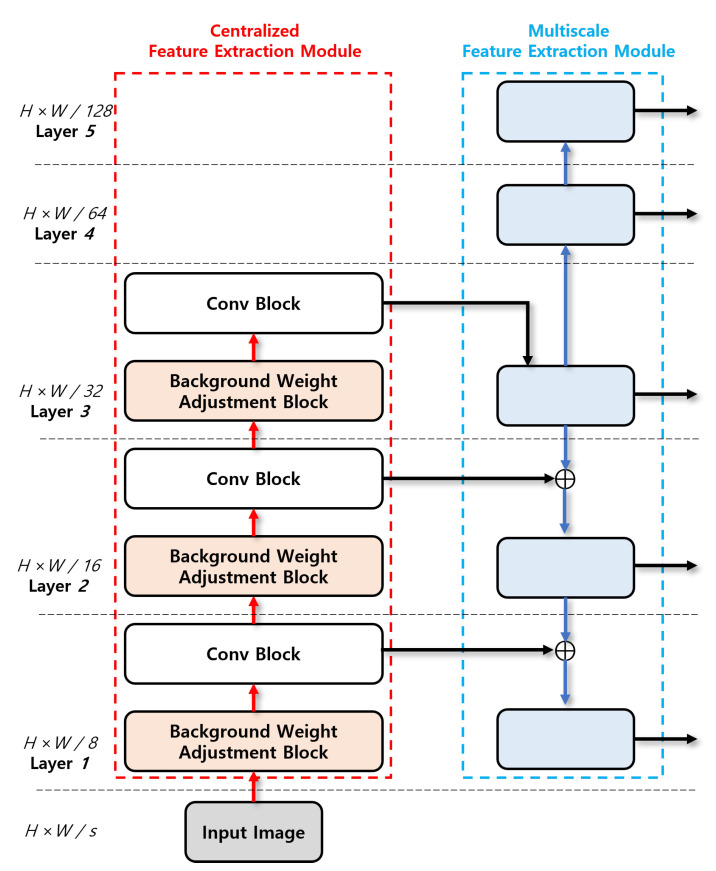
Centralized feature extraction module.

Then, the function of Background Weight Adjustment kernel is defined as follows:

(1) $$K_{back}\left(x,\;y\right)=exp(-(|\frac{x}{\sigma }\left|+\right|\frac{y}{\sigma }{\left|\right)}^{\gamma }),$$

where σ is the scale factor for the area of the kernel, and γ is the scale factor for the gradient of the kernel. Following the bottom-up pathway, centralized feature extraction module reduces the size of the feature map through downsampling by half at each layer for the input image, and creates different feature maps with various scales, which are transferred to multiscale feature extraction module. Regarding Figure 2, *H* and *W* are the height and width of feature maps, respectively. *s* is the downsampling ratio of the feature maps.

#### 3.1.2. Multiscale Feature Extraction Module

**Figure 3:**
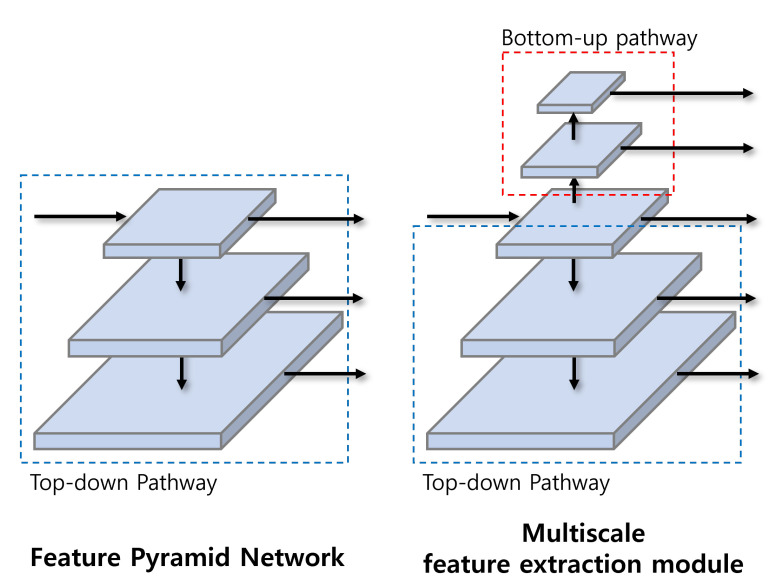
Multiscale feature extraction module and feature pyramid network.

The multiscale feature extraction module generates feature maps of different scales through convolution layer based on feature map received from the backbone. The multiscale feature extraction module consists of two pathways: bottom-up and top-down. The bottom-up pathway reduces the size of the feature map by half through downsampling, and the top-down pathway doubles the size of the feature map through upsampling. The previous FPN produces three feature maps using only the top-down pathway as shown in Figure 3. Our multiscale feature extraction module provides consistent detection accuracy by adding the bottom-up pathway to FPN and thus generating five feature maps of different sizes with level from layer 1 through layer 5.

#### 3.1.3. Prediction Module

There are five prediction modules each of which performs classification and regression simultaneously for object instance. They efficiently find the small-sized wastes by using anchor-free detection, and improve detection accuracy by exploiting the feature maps of different size. Prediction module consists of two blocks: OISB (Object Instance Separation Block) and ODB (Object Detection Block). OISB reduces the interference between adjacent objects by reducing the edge weight of each object through instance separation kernel. Let $b_{l}\;=\;\left[bx_{l},\;by_{l},\;w_{l},\;h_{l}\right]$ be the bounding box information, where *i* denotes a layer level of prediction module, and $\left(bx_{l},\;by_{l}\right)$ is the center coordinate of bounding box $b_{l}$, and $\left(w_{l},\;h_{l}\right)$ is the width and height of bounding box, respectively. Let (x,y) be the coordinates of bounding box with $\left(bx_{l},\;by_{l}\right)$ as origin, and $x\;\in \;X\;=\;\left\{-w_{l}/2,\;\ldots ,\;w_{l}/2\right\},\;\;y\;\in \;Y\;=\;\left\{-h_{l}/2,\;\ldots ,\;h_{l}/2\right\}$.

Then, Instance Separation Kernel is defined as follows:

(2) $$K_{instance}\left(x,\;y\right)=\frac{1}{2\pi \sigma }exp\left(-\frac{x^{2}\;+\;y^{2}}{2\sigma }\right),\;\sigma \;=\;3$$

**Figure 4:**
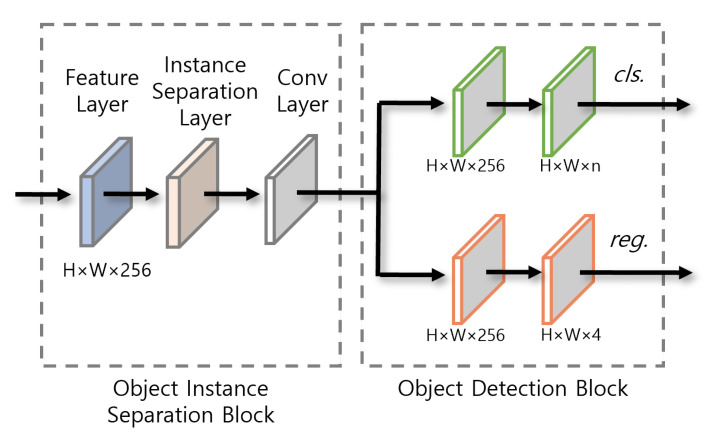
Prediction module.

Feature layer receives the feature map from multiscale feature extraction module, and then instance separation layer adjusts the edge weight of each object using the instance separation kernel in order to improve detection accuracy for overlapped multiple wastes of different types. The feature map is sent to ODB after applying the convolution layer. This phase is illustrated in Figure 4.

ODB detects multiple wastes with various sizes through anchor-free based object detection. Since the size of the anchor is fixed in the existing anchor-based models, it is difficult to detect the object smaller than the size of the anchor. ODB in our model is trained using the ground truth bounding box for the object instead of the fixed anchor most approximate to the bounding box in order to efficiently find the small objects whose bounding boxes are much smaller than the anchor candidate. ODB finds the loss value for our model by using Class Loss Function (CLF) and Box Regression Loss Function (BRLF). CLF calculates a class loss for the object instance based on the focal loss function. It decreases the loss for the high probability class while increasing for the low probability class. Let *p* be the predicted probability of the class for the object instance. Given *p*, CLF is defined as follows:

(3) $$CLF\left(p\right)\;=\;-{\left(1\;-\;p\right)}^{\gamma }log\left(p\right),\;\gamma \;=\;2,$$

where γ is the adjustable parameter.

BRLF is a bounding box regression function which calculates a loss by finding the IoU between the ground truth bounding box and the prediction bounding box.

Let $\tilde{x}\;=\;\left(x_{1}^{g},\;y_{1}^{g},\;x_{2}^{g},\;y_{2}^{g}\right)\;\in \;R^{4}$ be the coordinates of the ground truth bounding box for the object instance, and $\left(x_{1}^{g},\;y_{1}^{g}\right)$ and $\left(x_{2}^{g},\;y_{2}^{g}\right)$ the top-left and bottom-right corners of the ground truth bounding box, respectively. Let $x\;=\;\left(x_{1}^{p},\;y_{1}^{p},\;x_{2}^{p},\;y_{2}^{p}\right)\;\in \;R^{4}$ be the coordinates of the predicted bounding box of our model for the object instance, and $\left(x_{1}^{p},\;y_{1}^{p}\right)$ and $\left(x_{2}^{p},\;y_{2}^{p}\right)$ the top-left and bottom-right corners of the predicted bounding box, respectively. Given $\left(x,\;\tilde{x}\right)$, BRLF is defined as follows:

(4) $$\begin{array}{cc} & X\;=\;\left(x_{2}^{p}-x_{1}^{p}\right)\;*\;\left(y_{2}^{p}-y_{1}^{p}\right),\;\;\;\tilde{X}\;=\;\left(x_{2}^{g}-x_{1}^{g}\right)\;*\;\left(y_{2}^{g}-y_{1}^{g}\right)\\ & I\;=\;\left(min\left(x_{2}^{g},x_{2}^{p}\right)-max\left(x_{1}^{g},x_{1}^{p}\right)\right)*\left(min\left(y_{2}^{g},y_{2}^{p}\right)-max\left(y_{1}^{g},y_{1}^{p}\right)\right)\\ & U\;=\;X\;+\;\tilde{X}\;-\;I\\ & \mathrm{IoU}\;=\;I\;/\;U\\ & \mathrm{BRLF}(x\;,\;\tilde{x})\;=\;-\ln(\mathrm{IoU}) \end{array}$$

Then, the loss function *L* at layer *l* in the prediction module is defined as follows:

(5) $$L\left(l\right)\;=\;\frac{CLF_{l}\left(p\right)\;+\;BRLF_{l}\left(x,\tilde{x}\right)}{2}$$

Then, the final total loss $L(\hat{l})$ is defined as the minimum loss, where

(6) $$\hat{l}\;=\;\arg\;min_{l}\;L\left(l\right)$$

### 3.2. ARTD-Net2

**Figure 5:**
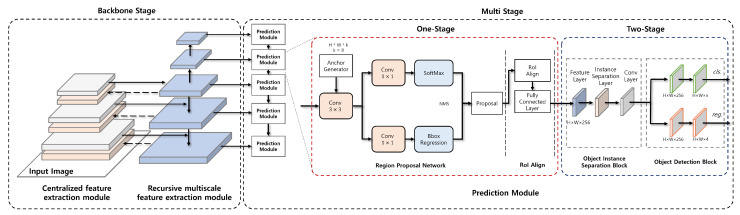
ARTD-Net2.

ARTD-Net2 is a multi-stage model which improves the accuracy of waste detection compared to ARTD-Net1 by attaching Region Proposal Network in the prediction module and additionally exploiting recursive feature extraction scheme as shown in Figure 5. ARTD-Net2 is slower to detect than ARTD-Net2, but ARTD-Net2 has higher detection accuracy than ARTD-Net1.

**Figure 6:**
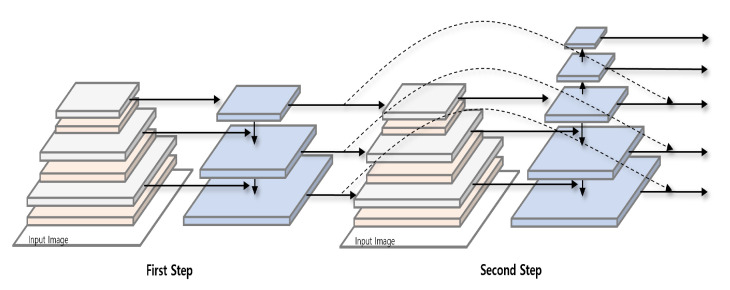
Recursive multiscale feature extraction module.

The centralized feature extraction module is used as the backbone in the multi-stage model. The feature map in each layer is sent to recursive multiscale feature extraction module connected to the layer as shown in Figure 6.

Recursive multiscale feature extraction module recursively performs the process of extracting features by multiscale feature extraction module to minimize feature loss. It improves accuracy by extracting important but missing features through the feedback network which sends the result of the first step to the input of the second step. The top-down pathway of multiscale feature extraction module is implemented as the unrolled iteration, and can be repeated n times.

The prediction module consists of two stages: the first stage has Region Proposal Network (RPN) and RoIAlign, and the second stage OISB and ODB. It is designed to combine anchor-based RPN and anchor-free ODB. RPN improves detection accuracy by increasing the number of anchor candidates. RoIAlign converts the results of RPN, which have different sizes, into a fixed-sized feature map in order to input them into the fully connected layer. OISB reduces the interference between nearby objects by reducing the edge weight of each object through instance separation kernel. ODB improves accuracy by detecting small objects which are not detected in RPN through anchor-free-based object detection.

## 4. Experiments

In this section, we shall describe about the performance evaluation for ARTD-Net1 and ARTD-Net2, respectively. Our experimental environment consists of eight GPUS, each NVIDIA A100 40 GB, and one AMD EPYC Processor with 92 single-cores and 1.7TB RAM. Our model was implemented using the Pytorch framework and MMDetection [41] with Python. We use Stochastic Gradient Descent (SGD) as the optimizer and the initial learning rate was set at 0.01. Weight decay of 0.0001 and momentum of 0.9 were used. The batch size was set at 32 (four images per GPU). The parameters σ and γ of Background Weight Adjustment kernel were set to 0.85 and 10, respectively. We use official codes downloaded from MMdetection for performance comparison of our models, and fine-tuned the parameters following the rules presented in the respective papers to achieve the best performance. In the case of YOLOv5 and YOLOv6, we downloaded the models from the official GitHub of authors. All models presented in this paper were trained on the recyclables dataset.

We used F1 score and mean Average Precision (mAP) as evaluation metrics to measure the performance of model. The F1 score is the harmonic mean of precision and recall, and is particularly useful for evaluating model performance on datasets with class imbalance. mAP is evaluation metric in object detection tasks. It is calculated as the mean of the Average Precision (AP) scores for each class.

This section is organized as follows: Section 4.1 describes the recyclables dataset used in the experiments in detail. Section 4.2 evaluates the performance of ARTD-Net1 and ARTD-Net2, respectively.

### 4.1. Recyclables Dataset

We build our recyclables dataset by focusing on the overlapped multiple wastes of different types with the complex arrangement. It comprises a total of 50,183 images of various resolutions and 110,759 annotation data, and has the largest number of annotations among trash datasets. The resolution of the image is 1280 × 720 on average and 4032 × 2268 at maximum. Several types of waste, up to eight, are placed in one image in order to increase the batch complexity over the previous dataset. Recyclable waste objects are classified into ten classes: paper, paper pack, paper cup, can, bottle, PET, plastic, vinyl, cap, label. Our dataset format follows COCO. Table 1 shows the number of annotations for each class of training and validation dataset.

The cap class has the maximum number of 8462 data and the paper pack class has the second largest number of 7705 data. Cap and label classes have the largest number of annotations as they are obtained from PET or bottles.

**Figure 7:**
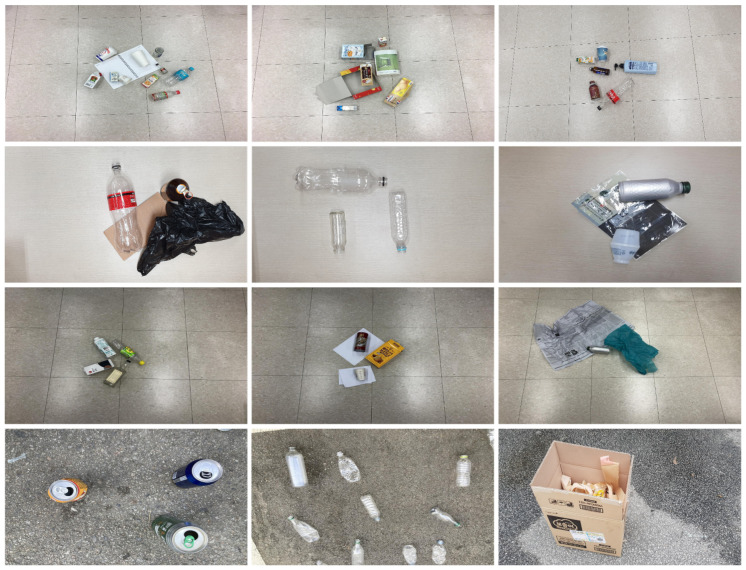
Sample images of dataset.

As shown in Figure 7, we collect overlapped multiple wastes of different types in various backgrounds and lighting conditions to make the deep learning model robust.

### 4.2. Performance Evaluation

#### 4.2.1. ARTD-Net1

We make use of our dataset in the previous section for the experiments. Experiments are conducted for F1 Score and mAP using the same backbone, ResNet-50. As shown in Table 2, we compare mAP according to the size of the object: small, medium and large.

First, we investigate the performance of centralized feature extraction module with BWAB in the backbone. It shows that adding BWAB into backbone improves the accuracy by reducing the edge weights around the border for the image. As shown in Table 2, our model with BWAB has higher F1 score and mAP than that without BWAB, showing better performance. Our model has F1 score of 82.043 and mAP of 0.495. It has higher F1 score of 2.12 and mAP of 0.027 than the model without both blocks.

Second, we check the performance of prediction module. It shows that adding OISB into prediction module improves the accuracy by reducing the edge weight of each object. our model with OISB has higher F1 score and mAP than that without OISB, showing better performance. Our model has F1 score of 81.688 and mAP of 0.506. It has higher F1 score of 1.765 and mAP of 0.038 than the model without both blocks.

Our model with both of BWAB and OISB has the highest score for F1 score and mAP.

We compare the F1 score and mAP of ARTD-Net1 with the other one-stage models. As shown in Table 3, we compare F1 score and mAP according to the size of the object: small, medium and large. For F1 score and mAP, ARTD-Net1 with ResNext-101 [42] achieves the highest performance. Among the models excluding ARTD-Net1, YOLOv6 with CSPStackRep as the backbone achieved the highest F1 score, and YOLOX-X with Modified CSPDarknet v5 as the backbone showed the highest mAP. ARTD-Net1 with ResNext-101 has higher F1 score and mAP of 0.275 and 0.004, respectively, than those of YOLOX-X with Modified CSPDarknet v5 as backbone. ARTD-Net1 with ResNext-101 has higher F1 score and mAP of 0.231 and 0.007, respectively than those of YOLOv6 with CSPStackRep as backbone. Among models excluding YOLO series, CornerNet with HourGlass-104 [43] as a backbone has the highest F1 score of 84.724 and CentripetalNet with HourGlass-104 as a backbone has the highest mAP of 0.586. ARTD-Net1 shows the best performance among the one-stage models.

#### 4.2.2. ARTD-Net2

We make use of our dataset for the experiments. Experiments are conducted for F1 Score and mAP using the same backbone, ResNet-101. As shown in Table 4, we compare mAP according to the size of the object: small, medium and large.

First, we investigate the performance of centralized feature extraction module with BWAB in the backbone of ARTD-Net2. It shows that adding BWAB into backbone improves the accuracy by reducing the edge weights around the border for the image. As shown in Table 4, our model with BWAB has higher F1 score and mAP than that without BWAB, showing better performance. Our model has F1 score of 85.31 and mAP of 0.57. It has higher F1 score of 1.44 and mAP of 0.03 than the model without both blocks.

Second, we check the performance of prediction module of ARTD-Net2. It shows that adding OISB into prediction module improves the accuracy by reducing the edge weight of each object. Our model with OISB has a higher F1 score and mAP than that without OISB, showing better performance. Our model has an F1 score of 85.14 and mAP of 0.581. It has a higher F1 score of 1.27 and mAP of 0.041 than the model without both blocks.

For all the cases, F1 Score and mAP achieve the highest score when using both BWAB and OISB.

We compare the F1 score and mAP of ARTD-Net2 with the other multi-stage models. As shown in Table 5, we compare F1 score and mAP according to the size of the object: small, medium and large. For all the cases, ARTD-Net2 with ResNeXt-101 achieves the highest performance. Among the models excluding ARTD-Net2, DetectoRS with ResNeXt-101 as the backbone has the highest performance. ARTD-Net2 with ResNeXt-101 has higher F1 score and mAP of 2.105 and 0.005, respectively than those of DetectoRS with ResNeXt-101 as backbone.

#### 4.2.3. Performance Comparison between ARTD-Net1 and ARTD-Net2

We analyze the two experimental results to compare the performance of ARTD-Net1 and ARTD-Net2: Confusion Matrix, Processing Time and Accuracy.

**Figure 8:**
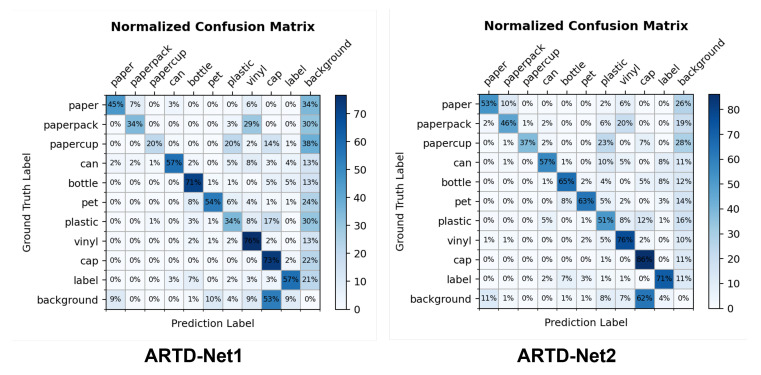
Confusion matrix of ARTD-Net1 and ARTD-Net2.

**Figure 9:**
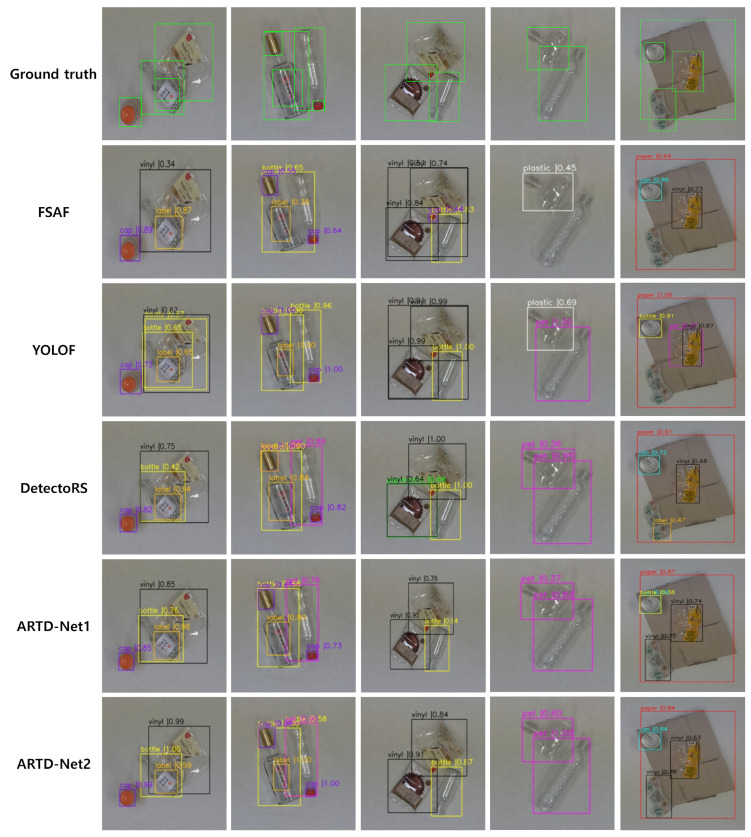
Inference result of ARTD-Net1, ARTD-Net2 and existing deep learning model.

First, we compare the accuracy of each class by using the confusion matrix after training our model with dataset. Figure 8 shows the accuracy of each class on ARTD-Net1 and ARTD-Net2 when using ResNet-101 as a backbone. According to the confusion matrix, ARTD-Net has significantly higher accuracy for bottle and vinyl. Those two components are not difficult to detect, since their features are clear. Large-sized recyclables are generally well detected. However, in the case of cap, false positives occur frequently, since they are small and similar to the background. In particular, the paper cup class is one of the most difficult to detect among the recyclable classes. The accuracy for paper cup class on ARTD-Net1 is 20%, while the accuracy for paper cup class on ARTD-Net2 is 37%, resulting in an improvement in performance. The inference results of ARTD-Net1 and ARTD-Net2 are shown in Figure 9.

**Figure 10:**
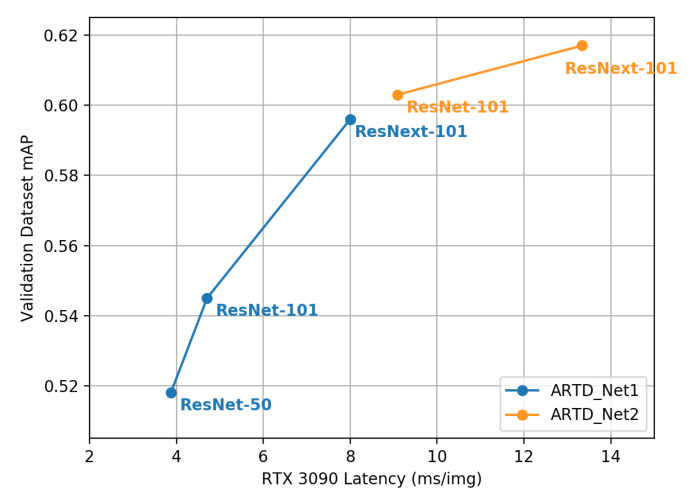
Processing time (ms/img) and accuracy (mAP) using recyclables dataset on ARTD-Net1 and ARTD-Net2.

Second, we compare the performance of ARTD-Net1 and ARTD-Net2 through accuracy and processing time of our model. As shown in Figure 10, ARTD-Net1 with ResNet-50 achieves the fastest processing time. ARTD-Net2 with ResNeXt-101 as the backbone has the highest performance. ARTD-Net2 with ResNet-101 has higher accuracy than ARTD-Net1 with ResNeXt-101. However, ARTD-Net1 with ResNeXt-101 has faster processing speed than ARTD-Net2 with ResNet-101.

Finally, we show that ARTD-Net1 is faster than ARTD-Net2 but ARTD-Net2 is more accurate than ARTD-Net1.

## 5. Conclusions

In this paper, we have proposed two anchor-free-based Recyclable Trash Detection Networks (ARTD-Net) which can recognize overlapped multiple wastes of different types efficiently by using edgeless module: ARTD-Net1 and ARTD-Net2. The former is an anchor-free based one-stage deep learning model which consists of three modules: centralized feature extraction, multiscale feature extraction and prediction. The centralized feature extraction module in the backbone architecture is obtained by adding BWAB to RetinaNet. BWAB exploits a new scheme which efficiently extracts features around the center of the input image while reducing the edge weights around the border of the image. The multiscale feature extraction module provides feature maps of different scales through bottom-up and top-down pathways. The prediction module consists of two blocks: OISB and ODB. OISB reduces the interference between nearby objects by reducing the edge weight of each object through instance separation kernel. ODB improves accuracy by detecting small objects which are not detected in RPN through anchor-free-based object detection. The latter is an anchor-free based multi-stage deep learning model which can efficiently finds each of waste regions by additionally exploiting Region Proposal Network and RoIAlign. It sequentially performs classification and regression to improve accuracy. We have presented a new recyclables dataset which is composed of a large number of high-resolution waste images with additional essential classes. We demonstrated that waste detection performance is improved by providing various images with the complex arrangement of overlapped multiple wastes with different types. The performance of ARTD-Net is confirmed through various experiments. ARTD-Net1 with BWAB and OISB achieves F1 score and mAP of 82.849 and 0.518, respectively, which is 2.926 and 0.05 higher than ARTD-Net1 without BWAB and OISB. Among the one-stage models, ARTD-Net1 with ResNext-101 achieves the highest F1 score and mAP of 85.594 and 0.596, respectively. ARTD-Net2 with BWAB and OISB achieves F1 score and mAP of 86.089 and 0.603, respectively, which is 2.219 and 0.063 higher than ARTD-Net2 without BWAB and OISB. Among the multi-stage models, ARTD-Net2 with ResNext-101 achieves the highest F1 score and mAP of 88.351 and 0.617, respectively. Finally, we demonstrated that ARTD-Net1 is faster than ARTD-Net2 but ARTD-Net2 is more accurate than ARTD-Net1.

For future works, we shall work on the details of ARTD-Net such as the number of blocks for each module and hyperparameters, and continue to collect data under more various lighting and background conditions to use our dataset for various works such as field diagnostics.

## Figures and Tables

**Table 1:** Annotation data for each category and class.

Category	Paper	Paper Pack	Paper Cup	Can	Bottle	PET	Plastic	Vinyl	Cap	Label
Train	9208	15,364	7867	8661	9075	5593	12,759	9468	18,043	13,419
Validation	183	76	65	139	145	122	137	152	112	170
Total	9391	15,440	7932	8800	9220	5715	12,896	9620	18,155	13,589

**Table 2:** Performance of ARTD-Net1, S: small, M: medium, L: large.

Model	F1 Score	mAP	mAP 50	mAP 75
		mAP S	mAP M	mAP L
Without BWAB and OISB	79.923	0.468	0.661	0.541
		0.145	0.397	0.561
With BWAB Without OISB	82.043	0.495	0.677	0.564
		0.159	0.402	0.573
Without BWAB With OISB	81.688	0.506	0.663	0.565
		0.16	0.398	0.593
With BWAB and OISB	82.849	0.518	0.678	0.585
		0.159	0.426	0.625

**Table 3:** Comparison between ARTD-Net1 and other one-stage deep learning models.

Model	Backbone	F1 Score	mAP	mAP50	mAP75	mAPs	mAPm	mAPl
SSD300	VGG-16	75.573	0.307	0.511	0.326	0.045	0.255	0.377
SSD512	VGG-16	75.952	0.338	0.543	0.357	0.051	0.29	0.401
RetinaNet	ResNet-50	77.161	0.353	0.567	0.384	0.031	0.308	0.481
RetinaNet	ResNet-101	78.208	0.373	0.58	0.4	0.048	0.325	0.507
FCOS	ResNet-50	81.46	0.52	0.698	0.577	0.144	0.467	0.612
FCOS	ResNet-101	82.623	0.525	0.695	0.582	0.17	0.435	0.629
FSAF	ResNet-50	80.212	0.479	0.657	0.539	0.147	0.406	0.564
FSAF	ResNet-101	80.63	0.496	0.683	0.573	0.165	0.432	0.595
SABL	ResNet-50	79.805	0.475	0.624	0.531	0.155	0.386	0.592
SABL	ResNet-101	80.083	0.513	0.642	0.57	0.175	0.397	0.605
CornerNet	HourGlass-104	84.724	0.575	0.711	0.631	0.181	0.517	0.636
CentripetalNet	HourGlass-104	79.326	0.586	0.742	0.639	0.198	0.502	0.657
SOLOv2	ResNet-50	80.71	0.485	0.673	0.533	0.109	0.392	0.59
SOLOv2	ResNet-101	81.634	0.525	0.705	0.572	0.127	0.424	0.619
YOLOv3	DarkNet-53	71.379	0.239	0.411	0.25	0.046	0.175	0.367
YOLOv4	CSP-Darkent53	81.272	0.499	0.652	0.544	0.148	0.396	0.655
YOLOv5	Modified CSP v5	84.762	0.587	0.737	0.619	0.198	0.471	0.663
YOLOF	ResNet-50	78.203	0.355	0.513	0.37	0.169	0.312	0.467
YOLOX-X	Modified CSP v5	85.319	0.592	0.778	0.669	0.19	0.508	0.699
YOLOv6	CSPStackRep	85.363	0.589	0.761	0.681	0.197	0.49	0.716
ARTD-Net1	ResNet-50	82.849	0.518	0.678	0.585	0.159	0.426	0.625
ARTD-Net1	ResNet-101	84.899	0.545	0.711	0.621	0.193	0.439	0.666
ARTD-Net1	ResNeXt-101	85.594	0.596	0.775	0.677	0.178	0.483	0.733

**Table 4:** Performance of ARTD-Net2, S: small, M: medium, L: large.

Model	F1 Score	mAP	mAP 50	mAP 75
		mAP S	mAP M	mAP L
Without BWAB and OISB	83.87	0.54	0.697	0.61
		0.163	0.434	0.624
With BWAB Without OISB	85.31	0.57	0.742	0.635
		0.182	0.445	0.638
Without BWAB With OISB	85.14	0.581	0.756	0.62
		0.194	0.462	0.645
With BWAB and OISB	86.089	0.603	0.761	0.646
		0.211	0.479	0.652

**Table 5:** Comparison between ARTD-Net2 and other multi-stage deep learning models.

Model	Backbone	F1 Score	mAP	mAP50	mAP75	mAPs	mAPm	mAPl
Faster R-CNN	ResNet-101	78.149	0.488	0.638	0.534	0.136	0.386	0.643
Faster R-CNN	ResNeXt-101	80.733	0.521	0.677	0.588	0.167	0.418	0.66
Cascade R-CNN	ResNet-101	81.373	0.529	0.686	0.575	0.152	0.41	0.653
Cascade R-CNN	ResNeXt-101	83.568	0.546	0.699	0.596	0.174	0.433	0.68
Double-Head R-CNN	ResNet-101	82.841	0.57	0.71	0.611	0.172	0.442	0.649
Double-Head R-CNN	ResNeXt-101	84.779	0.583	0.753	0.643	0.195	0.486	0.664
Sparse R-CNN	ResNet-101	83.717	0.578	0.715	0.638	0.176	0.476	0.655
Sparse R-CNN	ResNeXt-101	85.635	0.593	0.766	0.653	0.208	0.491	0.673
DetectoRS	ResNet-101	84.9	0.58	0.744	0.647	0.194	0.489	0.676
DetectoRS	ResNeXt-101	86.246	0.612	0.782	0.659	0.224	0.507	0.686
ARTD-Net2	ResNet-101	86.089	0.603	0.761	0.646	0.211	0.479	0.652
ARTD-Net2	ResNeXt-101	88.351	0.617	0.806	0.665	0.233	0.511	0.711

## Data Availability

The data presented in this study are available on request from the corresponding author.
